# A Longitudinal Study of Motor, Oculomotor and Cognitive Function in Progressive Supranuclear Palsy

**DOI:** 10.1371/journal.pone.0074486

**Published:** 2013-09-10

**Authors:** Boyd C. P. Ghosh, Roger H. S. Carpenter, James B. Rowe

**Affiliations:** 1 Wessex Neuroscience Centre, Southampton University Hospital NHS Trust, Southampton, Hampshire, United Kingdom; 2 Department of Clinical Neurosciences, Cambridge University, Cambridge, Cambridgeshire, United Kingdom; 3 Department of Physiology, Development and Neuroscience, University of Cambridge, Cambridge, Cambridgeshire, United Kingdom; 4 MRC Cognition and Brain Sciences Unit, Cambridge, Cambridgeshire, United Kingdom; 5 Behavioural and Clinical Neuroscience Institute, Cambridge, Cambridgeshire, United Kingdom; Oslo University Hospital, Norway

## Abstract

**Objective:**

We studied the annual change in measures of motor, oculomotor and cognitive function in progressive supranuclear palsy. This had twin objectives, to assess the potential for clinical parameters to monitor disease progression in clinical trials and to illuminate the progression of pathophysiology.

**Methods:**

Twenty three patients with progressive supranuclear palsy (Richardson’s syndrome) were compared to 22 matched controls at baseline and 16 of these patients compared at baseline and one year using: the progressive supranuclear palsy rating scale; the unified Parkinson’s disease rating scale; the revised Addenbrooke’s cognitive examination; the frontal assessment battery; the cubes section of the visual object and space perception battery; the Hayling and Brixton executive tests; and saccadic latencies.

**Results:**

Patients were significantly impaired in all domains at baseline. However, cognitive performance was maintained over a year on the majority of tests. The unified Parkinson’s disease rating scale, saccadic latency and progressive supranuclear palsy rating scale deteriorated over a year, with the latter showing the largest change. Power estimates indicate that using the progressive supranuclear palsy rating scale as an outcome measure in a clinical trial would require 45 patients per arm, to identify a 50% reduction in rate of decline with 80% power.

**Conclusions:**

Motor, oculomotor and cognitive domains deteriorate at different rates in progressive supranuclear palsy. This may be due to differential degeneration of their respective cortical-subcortical circuits, and has major implications for the selection of outcome measures in clinical trials due to wide variation in sensitivity to annual rates of decline.

## Introduction

In 1964 Drs Steele, Richardson and Olszewski published their seminal report of 9 patients “who displayed an unusual progressive neurological disorder with ocular, motor and mental features” [1], a condition now known as progressive supranuclear palsy (PSP) or Richardson’s syndrome. Subsequent work has sought to understand the natural history of the disease, including increased awareness of cognitive decline. With the advent of clinical trials of potential disease modifying drugs, many based on interfering with the hyperphosphorylation and aggregation of Tau protein, there is renewed interest in identifying reliable clinical markers of disease for early diagnosis and disease progression.

However, tests which are sensitive to the presence of the disease, may not be optimal for monitoring progression and vice versa [2]. Some investigators have proposed brain imaging as a biomarker [3,4]. Others have focussed on the clinical progression of PSP, either as part of a composite test such as the PSP rating scale [4–6], or with separate systems as summarised in Table 1. Some domains, such as motor ability, have been studied longitudinally using validated scales [7]. However, few studies have investigated the longitudinal change in other domains such as cognition. Predicting PSP tau pathology is possible with a high degree of accuracy in the presence of a typical PSP-RS (Richardson’s syndrome) phenotype [7], but without data detailing progression of disease in multiple domains, improvements in the rate of decline of patients in trials could be missed.

**Table 1:** Summary of studies giving data concerning the onset or progression of clinical aspects of PSP.

		**Motor**				**Oculomotor**		**Cog**	**Bulbar**			
		**b/kin**	**falls**	**w/chair**	**other**	**VSNGP**	**other**		**d/arthria**	**unintel**	**d/phagia**	**sev d/phagia**
**Brusa et al.[40]**	P					50% @ 2-4						
**Golbe et al.[41]^a^**	R			8.2	+		+		3.4		4.4	
**Pillon et al.[26]^b^**	P							+				
**Collins et al.[42]**	R		0.7 (0-3)			1.5 (0.5-2.5)						
**Litvan et al.[24]^c^**	R	88% [1] 96% [2]	83% [1] 100% [2]			79% [1]83% [2]		+	75% [1] 100% [2]		16% [1] 83% [2]	
**Santacruz et al.[27]^d^**	P	4.6-5.6	2.6-3.6	5.6-6.6			+	+	2.6-3.6	5.6-6.6	3.6-4.6	
**Muller et al.[30]**	R								2		3.5	
**Carrilho et al.[43]^e^**	M					+2.5 (0-5)			+1 (1–3)		+1(1-3)	
**Nath et al. [32]**	R		0 (0-16)						1.75 (0-15)		3.6(0-16)	5 (0.6-17)
**Goetz et al.[44]**	R			4.8 (3.8-7.6)						5.9 (4-8.3)		
**Macia et al.[25]^f^**	M		0.6 Daily 3.1			2.5+/-1	+	+	2	3.8	2.9	4.6
**Golbe et al.[5]^g^**	P			4.8-5.8	+							
**Donker Kaat et al.[21]**	P					3.9 (0-14)						
**O’Sullivan et al.[45]^h^**	R		3.9+/-2.5	6.4+/-2.7				+		6+/-2.5		6.4+/-2.4
**Bensimon et al.[7]^i^**	P				+							
**Payan et al.[6]^j^**	P				+		+	+				
**Whitwell et al.[4]^k^**	P				+							

Median time between disease onset and feature onset in years. Range is given in curved parentheses, standard deviation as +/-. P, R or M in the second column is prospective, retrospective or mixed prospective and retrospective studies respectively. A + in a column refers to the footnotes giving additional data or rates of progression. b/kin is bradykinesia, w/chair is when patients require a wheelchair, VSNGP is vertical supranuclear gaze palsy, d/arthria is dysarthria, unintel is unintelligible, d/phagia is dysphagia, sev d/phagia is severe dysphagia, cog is cognitive symptoms.

^a^ Gait difficulty was seen at 0.3 years and use of a walking aid at 3.1 years. Patients had visual symptoms at 3.9 years.

^b^ Neuropsychological diagnosis of dementia increased from 37.5% to 70% of patients in 15.3 months.

^c^ Frontal symptoms were noted in 46% [1] and 58% [2]. [1] refers to first visit and [2] to last visit. Mean time between visits was 2.2 years (+/- 1 year).

^d^ Time to not being able to read 2.6-3.6 years, word finding difficulties 3.6-4.6 years and memory problems 5.6-6.6 years. Times given are for 50% of patients to have the given level of difficulty or worse. First visit was at 2.6 years.

^e^ Times given here are times from diagnosis and marked with a +.

^f^ Figures refer to falls occurring at all or on a daily basis; some dysphagia and choking at each meal; blurred vision (1.2 years) and being unable to read (2.9 years). Cognitive difficulties were noted in 40% in the first two years and 88% in more advanced stages (mean 4.5 years).

^g^ Time given is the time from onset until less than 50% of patients with a baseline score of 40-49 retain some useful gait (PSPRS question 26 score < 4). Overall mean progression of PSPRS was 11.3 points per year.

^h^ Falls refers to more than 2 falls in a year. Cognitive impairment occurred at 4.2 years +/-2.9 (ADL impairment due to cognition deficits).

^i^ Short Motor Disability Scale 3.1+/-4.4, Schwab and England Activities of Daily Living - 16.3+/-17.5, Hoehn and Yahr 0.5+/-0.8, Clinical global impression of Disease severity 0.7+/-0.7. Figures given are mean points changed per year +/- standard deviation.

^j^ The NNIPPS- Parkinson plus scale is a composite scale consisting of among others, motor, ocular and mental features designed for use in multi-system atrophy and PSP. This showed a total rate of change of 25.8 points per year. Motor ability and bradykinesia changed at 4.0-4.6 points per year, oculomotor ability at 2.2 points per year, bulbar function at 3.2 points per year, mental function at 2.1 points per year and urinary function at 1.0 points per year.

^k^ Annual change in the PSPRS for those diagnosed initially with only probable PSP was 18 points a year.

In this study, our aim was to assess the rate of decline of neuropsychological, motor and oculomotor functions. Our hypothesis was that these functions would all be abnormal at the time of diagnosis, but that they would deteriorate differentially. This comparison is not only relevant to our ability to study disease progression in the context of disease modifying treatments: it would also offer new insights into the pathophysiological progression of PSP.

## Subjects and Methods

### Ethics Statement

The Cambridgeshire research ethics committee approved this study, including the information sheet, consent documents and all tests to be carried out. All investigations were carried out with the adequate understanding and written consent of the participants involved in the research. The capacity of all patients was assessed by trained medical staff including a consultant neurologist. No patient was recruited to this study if they did not have the capacity to consent. Capacity was assessed and consent was obtained again after any interval in testing greater than six weeks. The patients’ family was included in the process at each stage and, although not necessary, their agreement to testing was also obtained.

### Participants

Twenty-three patients were recruited prospectively from a specialist neurological clinic for patients with PSP and related disorders, at Addenbrooke’s hospital between 2007 and 2009. Contemporary clinical diagnoses for possible or probable PSP were made by an experienced neurologist according to consensus criteria [8]. With subsequent information the diagnoses have been revised to probable or definite PSP as, to date, ten of the patients have undergone post mortem examination: all ten had PSP. The phenotype identified by our inclusion criteria corresponds closely to the PSP-RS ‘Richardson’s syndrome’ rather than other clinical manifestations of PSP pathology such as PSP-P. Baseline assessment was carried out at recruitment with interval testing as close to a year after baseline assessment as practicable.

At baseline, one patient (A) was unable to complete the saccadometry, two patients (B and C) with poor visual acuity undertook a non-visual subset of tests only, and two patients (D and E) failed to complete all of the neuropsychological tests due to fatigue or intercurrent illness. At interval testing, patient A was unable to complete the saccadometry but was able to complete all other tests, patients B, C and D were unable to complete testing due to intercurrent illness or fatigue and patient E died before the planned interval of 1 year. In addition a further 3 patients died before the end of the interval period. Sixteen patients had complete or near complete data sets at interval testing.

Twenty two age- and education- matched controls were recruited from the panel of volunteers at the MRC Cognition and Brain Sciences Unit (CBU) or from spouses of patients. Controls had normal hearing and corrected vision and did not have significant neurological or psychiatric comorbidity.

### Motor and cognitive testing

Motor function was assessed with section III of the Unified Parkinson’s Disease Rating Scale (UPDRS) [9] and the PSP rating scale (PSPRS) [5]. The PSPRS also includes sections for bulbar, oculomotor and personality changes. Scores for UPDRS and PSPRS were transformed by simple inversion so that high scores represented better function for ease of comparison across all tests. This was achieved by subtracting participant scores from the maximal test score (108 for UPDRS, 100 for the PSPRS total score and 5 for the PSPRS stage subscore).

Cognitive testing used the Addenbrooke’s Cognitive Examination – revised (ACE-R) [10], the Frontal Assessment Battery (FAB) [11], the cubes subsection of the Visual Object and Space Perception Battery (VOSP) [12], the Hayling test and Brixton test [13]. There is a timed component in the Hayling test. Given the bradyphrenia and bradykinesia evident in PSP, we used only the number of incorrect responses in the second section of the test, assessing unsuccessful inhibition by participants, without reference to timing of responses. Hayling A errors refers to participants choosing, incorrectly, a stereotyped ending for a sentence. Hayling B errors are answers, which although semantically related, are not stereotyped. Scores for correct answers were given so that higher scores represented better function.

### Saccadometry

Saccadometry was completed at baseline and interval. Saccades were measured using a head mounted binocular infra red sclerometer, recorded at 1kHz and low pass filtered at 250Hz, with 12 bit resolution [14]. It presents targets for the participants using low powered lasers mounted on the front and angled at +10°, 0 and -10° azimuth. The saccadometer uses a step task paradigm. After a random initial period between 0.5 and 1.0 seconds the central target is extinguished and simultaneously either the left or right target presented. The device measures the latency of the resulting saccade (time between the target moving and eyes starting to move). The device was automatically calibrated using a short series of presentations of the targets at the beginning of the session. Participants sat at a distance of 1.5 metres from a blank wall with the room darkened. Because the stimuli move exactly with the head, a bite bar is not required, leading to increased subject comfort.

After testing, the data were downloaded to a laptop and pre-processed using Latency Meter v 2.10 [14]. This contains an automated validation program that compares the log likelihood value for the position and velocity traces for each trial to the mean and standard deviation values for all the trials in the session. This is used to reject blinks, saccades in the wrong direction, and grossly abnormal traces using a rejection threshold for either the velocity or position traces. Saccade data were then analysed using SPIC software employing the LATER (Linear Approach to Threshold with Ergodic Rate) model [15–17] and reciprobit plots of response latencies.

**Figure 1:**
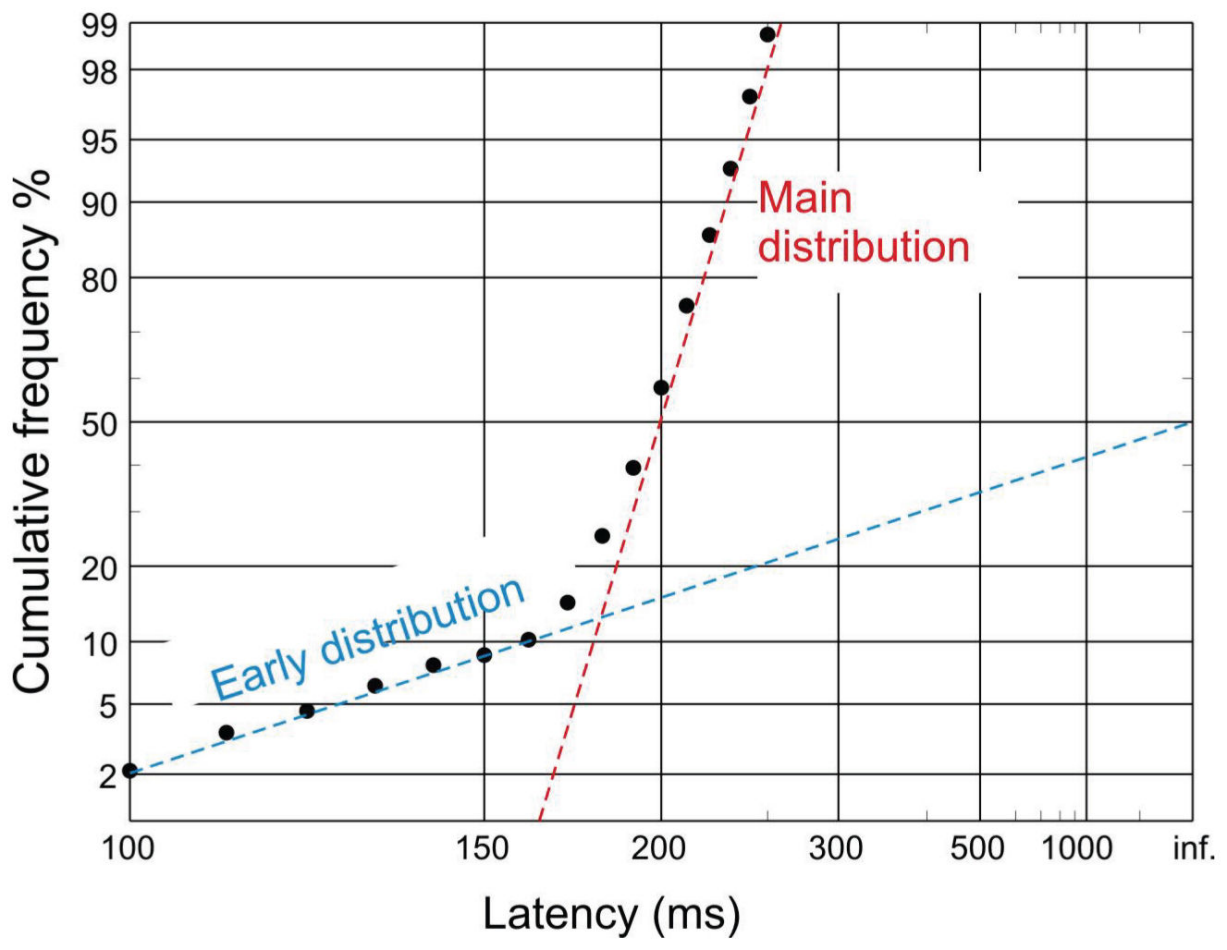
Reciprobit plot of saccade latencies from a healthy volunteer. Cumulative probability is plotted on the y axis on a probit scale. Using this scale, plotting a normal distribution results in a straight line. Latency is plotted on the x axis using a reciprocal scale. The reciprocals of the latencies are equally spaced along this scale. Additionally this scale is mirrored, so that short latencies are to the left and long to the right: infinite latencies, whose reciprocals are zero, therefore form the right hand margin. Because their reciprocal latencies are normally distributed, with mean mu and standard deviation sigma, most latencies lie on a straight line (red), the main distribution, whose median and slope correspond to mu and sigma. In addition, under some conditions there may be a sub-population of early saccades (blue) that lie on a line of shallower slope, corresponding to a third parameter early sigma.

A typical reciprobit plot of a series of latencies is shown in Figure 1. The majority of the saccade population adhere to a normal distribution of inverse latencies, and can be seen to lie along a straight line in the reciprobit plot. There are a minority of saccades that are generated differently, with a distinct normal distribution of inverse latencies with high variance. These are seen with a reduced latency and lie along a different line – the ‘early’ saccade distribution. Three parameters, the reciprocal of the median latency, mu, and the slopes of the early and main lines, early sigma and sigma respectively, are sufficient to describe the two inverse latency distributions and can be related directly to the physiology of visually evoked saccade generation [17]; these parameters are estimated from the observed distributions by minimisation of the Kolmogorov-Smirnov one-sample statistic.

### Statistical analysis

Statistical analysis used SPSS v 15 (SPSS Inc., Chicago, IL). Parametric data for patients and controls were compared with t-tests, one way (ANOVA) or repeated measures ANOVA (rm-ANOVA) with *post hoc* contrasts. Baseline and interval parametric data for patients were compared with paired t-tests. Non-parametric data were investigated with Mann Whitney or Kruskal Wallis. χ^2^ tests were used for categorical data.

Where the rate of change for different tests were compared, the annualised normalised score was used. This is the difference in scores for each test divided by the time interval between tests and multiplied by 1 year. This score was then divided by the maximum score for the test in order to compare different tests. For saccadometry, mu was divided by the mean plus two standard deviations for the controls (6.38).

Power calculations used Gpower 3.1.5 [18,19] with an alpha value of 0.05, Beta value of 0.2 (power 80%) and two sided t tests. Sample sizes were estimated for interventions that reduced the rate of decline by 25% and 50%.

## Results

**Figure 2:**
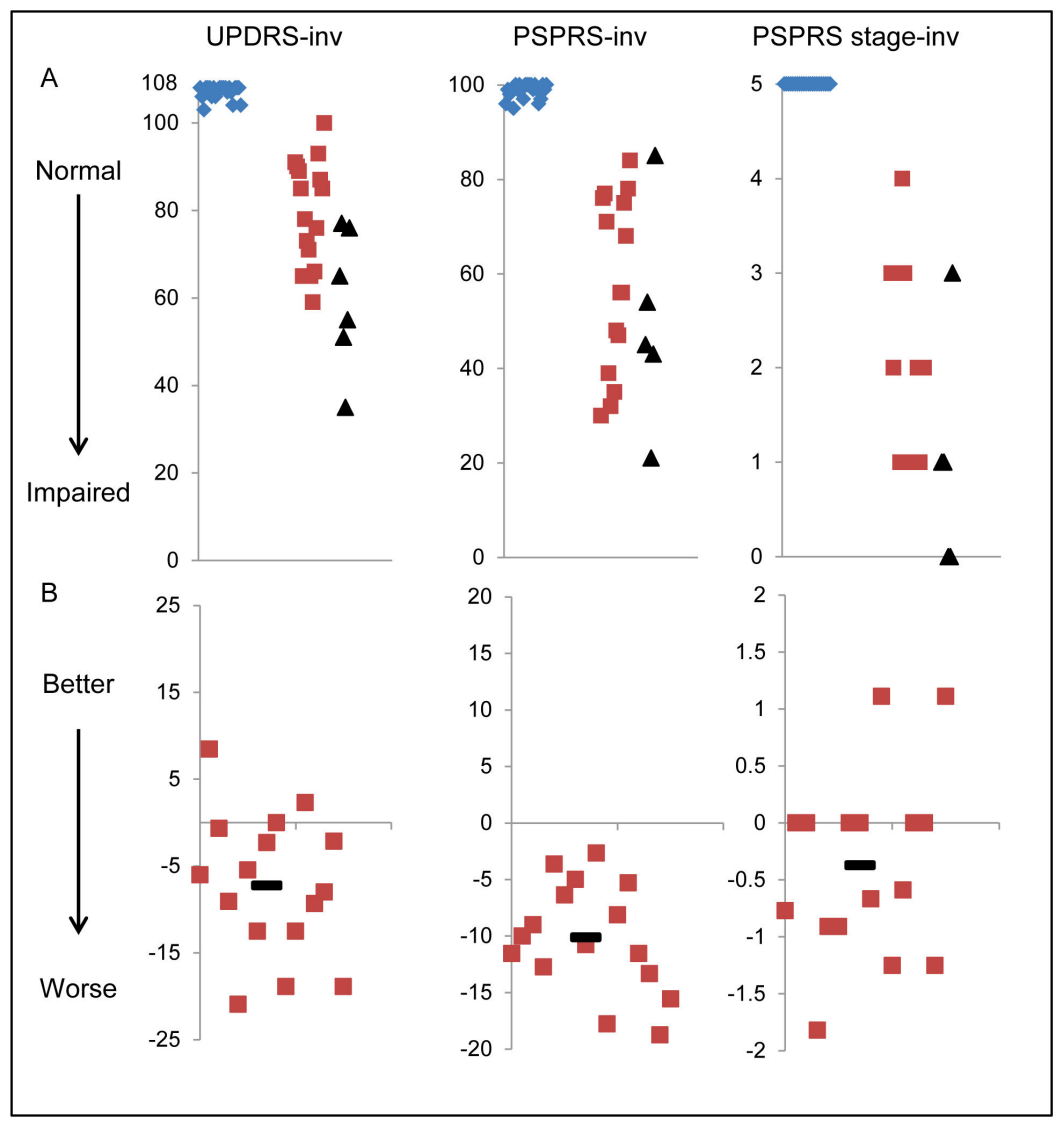
Baseline scores and annualised rates of change for motor tests. Row A shows baseline scores on each test. In order that all graphs show decline as lower values, the scores have been transformed by subtracting the participants’ score from the maximum for the test (see methods). Blue diamonds are controls, red squares are patients with PSP. Black triangles mark the baseline score for patients who could not complete interval testing. Row B shows the difference in score between baseline and interval, in those patients who completed both assessments. The score has been adjusted so that it shows the change in score over twelve months Negative values represent worsening of function. In both sets of graphs, the x axis represents a nominal value. In line A the x axis is arranged so that controls are on the left, patients who completed interval assessments are in the middle and patients who did not complete the interval assessment are on the right. In line B the scores are arranged randomly. Tests are named at the top of each column. UPDRS is the Unified Parkinson’s Disease Rating Scale motor sub scale; PSPRS is the PSP rating scale and PSPRS stage is the stage sub score of the PSPRS. All tests are marked “–inv” to signify that the scale for these tests has been inverted representing deficit from normal.

**Figure 3:**
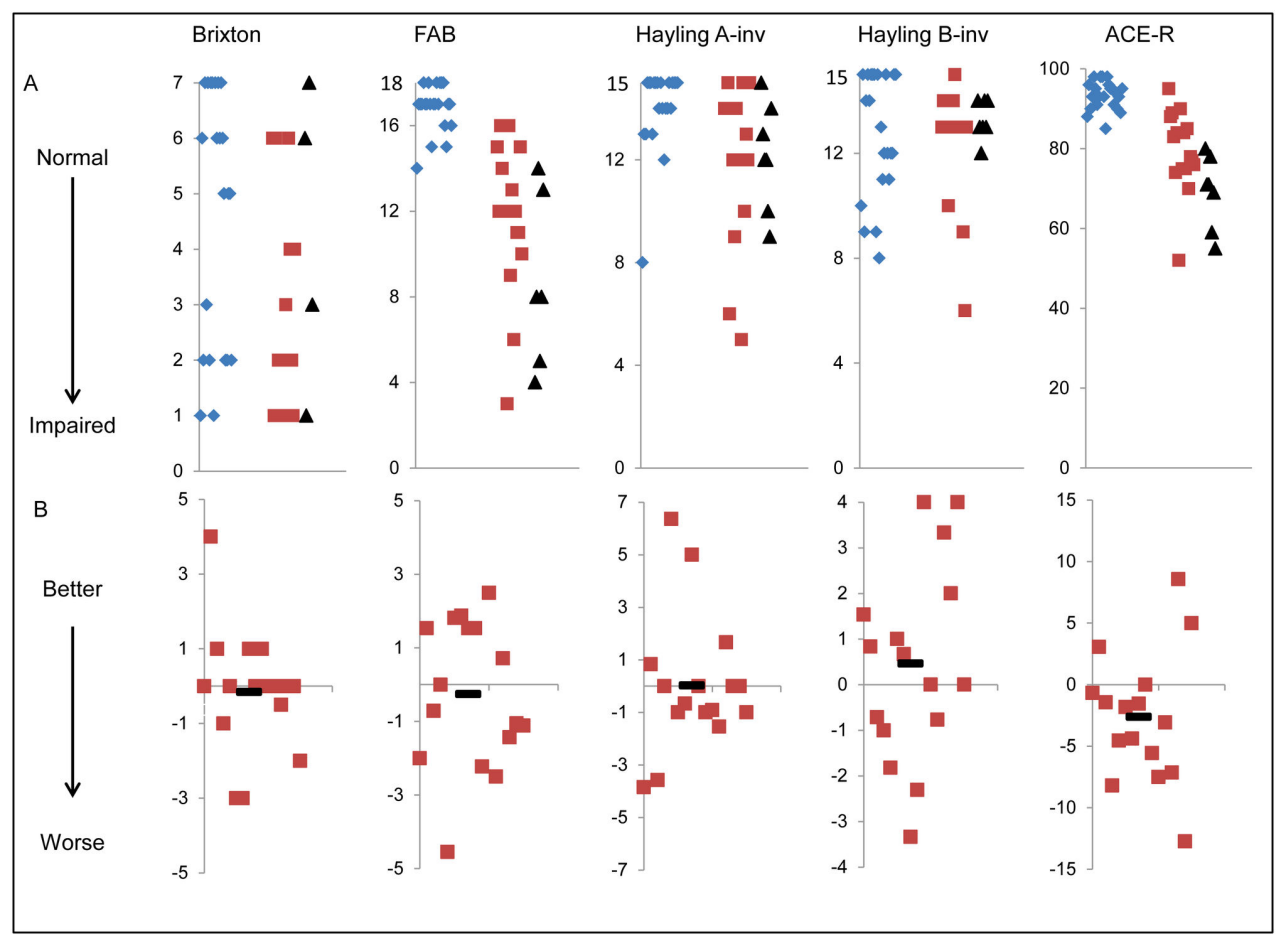
Baseline scores and annualised rates of change for cognitive tests. Tests are named at the top of each column. FAB is the Frontal Assessment battery; Hayling A-inv is the number of correct category A answers in the Hayling test; Hayling B-inv is the number of correct category B answers on the Hayling test; ACE-R is the Addenbrookes Cognitive Examination - Revised. Row A shows baseline scores on the test. Blue diamonds are controls, red squares are patients and black triangles mark the baseline score for patients who could not complete the interval testing. Hayling A and Hayling B scores have been transformed so that a higher score represents a better function (see methods). Row B shows the difference in score between baseline and interval, in those patients who completed both assessments. The score has been adjusted so that it shows the change in score over twelve months. Negative values represent worsening of function. In both sets of graphs, the x axis represents a nominal value. In line A the x axis is arranged so that controls are on the left, patients who completed interval assessments are in the middle and patients who did not complete the interval assessment are on the right. In line B the scores are arranged randomly.

**Figure 4:**
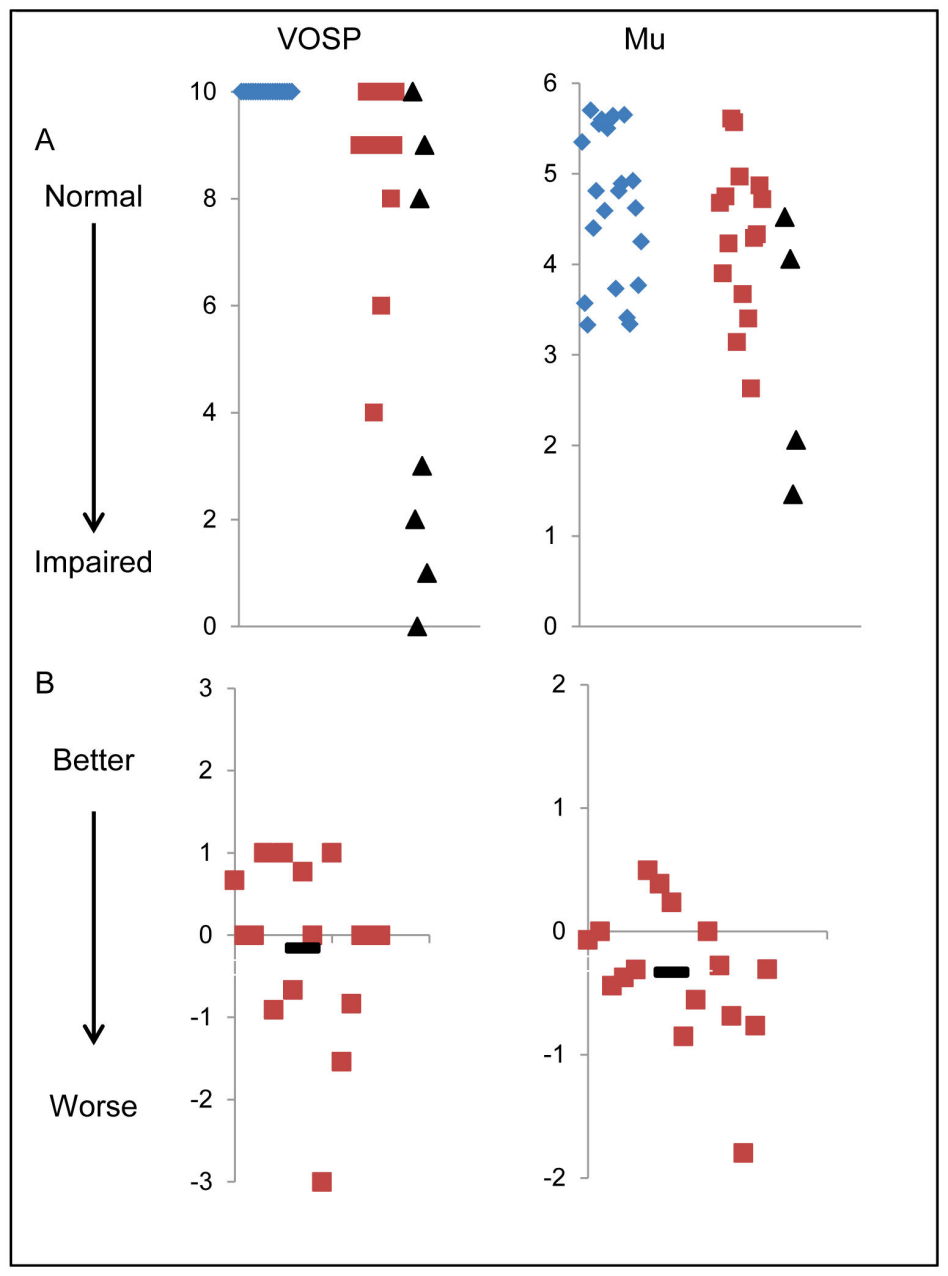
Baseline scores and annualised rates of change for visual and oculomotor tests. Tests are named at the top of each column. VOSP is the Visual Object and Space Perception battery and mu is the reciprocal of the latency as measured by saccadometry. Row A shows baseline scores on the test. Blue diamonds are controls, red squares are patients and black triangles mark the baseline score for patients who died before interval testing. A lower value on the y axis for the mu graph corresponds to a lengthening of latency between stimulus presentation and saccade initiation. Row B shows the difference in score between baseline and interval, in those patients who completed both assessments. The score has been adjusted so that it shows the change in score over twelve months, irrespective of how far apart the assessments were. Negative values represent worsening of function. In both sets of graphs, the x axis represents a nominal value. In line A the x axis is arranged so that controls are on the left, patients who completed interval assessments are in the middle and patients who did not complete the interval assessment are on the right. In line B the scores are arranged randomly.

The groups were well matched demographically at baseline (see table 2). Figures 2-4 show baseline test scores for controls and all patients. It can be seen that patients were significantly worse than controls at baseline for all tests (see also table 3). Repeat testing of the patients followed up were completed at a mean interval of 1.2 years (SE 0.07).

**Table 2:** Demographics and baseline scores for healthy controls and patients.

Group	N	Fem	AGE	EY	DD	O–D	UPDRS	PSPRS	Br	FAB	HayA	HayB	ACE	VOSP	Mu	Sigm
PSP Baseline	*23*	*39*	*71.1 (8.6)*	*11.0 (9.0-19.0)*	*3.0 (1.3-17.3)*	*2.2 (0.7-17)*	*33.8 (15.7)*	*45.0 (19.7)*	*3.1 (2.0)*	*10.8 (3.9)*	*3.0 (2.8)*	*2.3 (2.0)*	*76.4 (10.9)*	*7.6 (3.2)*	*4.0 (1.1)*	*1.3 (0.5)*
PSP Interval	16	40	68.6 (7.5)	11.0 (10.0-19.0)	4.0 (2.6-12.2)	2.2 (0.7-10.5)	28.4 (12.1)	43.3 (19.0)	2.8 (1.8)	11.7 (3.6)	3.0 (3.1)	2.5 (2.3)	79.7 (10.1)	8.9 (1.7)	4.3 (0.9)	1.4 (0.5)
PSP Deceased	4	50	72.3 (5.0)	10.5 (9-14)	4.6 (3.2-9.7)	2.5 (1.1-9.7)	56.5 (12.5)	63.7 (13.3)	3.7 (3.1)	7.8 (4.5)	4.0 (1.8)	1.8 (0.5)	69.3 (7.8)	5.5 (3.5)	3.0 (1.5)	1.2 (0.4)
PSP Unable	3	33	83 (9.0)	14 (9-19)	3 (1.8-17.3)	2.0 (1.0-17.0)	31.5 (0.7)	30.5 (21.9)	6.0	10.5 (3.5)	1.3 (1.5)	1.7 (1.2)	68.7 (12.7)	3.7 (5.5)	----	----
Controls	*22*	*32*	*71.4 (7.6)*	*13.0 (9.0-20.0)*	*N/A*	*N/A*	*1 (1.5)*	*1.3 (1.5)*	*4.7 (2.3)*	*16.9 (1.1)*	*1.0 (1.6)*	*2.2 (2.3)*	*93.4 (3.5)*	*10.0 (0)*	*4.7 (0.8)*	*1.0 (0.2)*

The groups consist of controls, all patients who were tested at baseline (PSP Baseline), baseline scores for those who were tested at both baseline and interval (PSP Interval), and baseline scores for those patients who were tested at baseline but either were unable to complete interval assessments (PSP Unable) or died before the interval assessments were due to be carried out (PSP Deceased). StD: standard deviation. N: number in the group, Fem refers to the percentage of females in the group, Br is Brixton test scaled score (there is no standard deviation given for “PSP unable” as there was only one patient score in this category), DD is the median symptomatic disease duration in years, EY is median education years, AGE is mean age, O–D is median time between onset and diagnosis in years, UPDRS is the unified Parkinson’s disease rating scale, PSPRS is the PSP rating scale, FAB is the frontal assessment battery, ACE-R is the Addenbrooke’s Cognitive Examination Revised, VOSP refers to the cubes subsection of the Visual Object and Space Perception battery, mu is the mean of the reciprocals of the median latency as measured by saccadometry and sigma is the mean of the variance of the saccade latencies. There are no values given for mu and sigma in “PSP unable” as the patients were unable to do the test at baseline. Hayling A refers to the number of category A errors in the Hayling test, and Hayling B to the number of category B errors. The scores for UPDRS, PSPRS, Hayling A and Hayling B are given untransformed, therefore a higher score indicates deteriorating function. All figures in parentheses are standard deviations except for those for EY, DD and O–D which are ranges.

**Table 3:** Test comparisons between controls and patients at baseline.

	**UPDRS**	**PSPRS**	**PSPRS stage**	**Brixton**	**FAB**	**Hayling A**	**Hayling B**	**ACE-R**	**VOSP**	**Mu**	**Sigma**
**Control Mean**	1	1.3	0	4.7	16.9	1.0	2.2	93.4	10.0	4.7	1.0
**Patient Mean**	33.8	45.0	3.3	3.1	10.8	3.0	2.3	76.4	7.6	4.0	1.3
**t(df)**	-9.7 (21.4)	-10.2 (20.2)	-13.7 (20.0)	2.3 (40)	6.8 (22.9)	-3.0 (35.7)	-0.12 (43)	7.1 (26.8)	3.6 (22)	2.1 (39)	-2.5 (24.3)
**P value**	<0.001	<0.001	<0.001	0.02	<0.001	0.005	1.0	<0.001	0.002	0.04	0.02

Mean control and patient baseline scores are given. UPDRS is the unified Parkinson’s disease rating scale, PSPRS is the PSP rating scale, FAB is the frontal assessment battery, ACE-R is the Addenbrooke’s Cognitive Examination revised, VOSP refers to the cubes subsection of the Visual Object and Space Perception battery, mu is the mean of the reciprocals of the median latency as measured by saccadometry and sigma is the mean of the variance of the saccade latencies. Hayling A refers to the number of category A errors in the Hayling test, and Hayling B to the number of category B errors. The scores for UPDRS, PSPRS, Hayling A and Hayling B are given untransformed, therefore a higher score indicates deteriorating function. t is the t statistic, df is the degrees of freedom, p values given are two tailed. Results have been corrected where control and patient variance were not equal. This process gives an altered value for degrees of freedom. Using Bonferroni correction with 10 comparisons, a p value less than p=0.005 would be significant. In that case mu, sigma, Hayling B and Brixton tests would not be significant.

Figures 2-4 and tables 4-6 show the annualised change in test score for those patients who completed both baseline and interval testing after a year with the paired t test comparison. It can be seen that for the majority of the tests, the mean change in score was either zero or very close to zero. The exceptions to this were the scores for the PSP Rating Scale (PSPRS) which showed a mean change over a year of 11.3 points (*t*(15)=11.2, *p*<0.001) (see table 4 and Figure 2), the Unified Parkinson’s Disease Rating Scale III (UPDRS), which showed a mean change of 8.3 points (*t*(15)=3.6, *p*=0.003) (see table 4 and Figure 2) and mu (the inverse median latency for saccades), which showed a mean decrease in mu of 0.4 seconds^-1^(equivalent to an increase in latency of 0.02 seconds) (*t*(13)=2.5, *p*=0.01) (see table 6 and Figure 4).

**Table 4:** Baseline and interval scores with paired t tests for motor tests.

	UPDRS*	PSPRS*	**PSPRS stage**
**Baseline**	28.4	32.8	3.1
**Interval**	36.7	44.1	3.6
**t (df)**	3.6 (15)	11.2 (15)	2.2 (15)
**p value**	0.003	<0.001	0.02

Mean baseline and interval scores are given. UPDRS is the unified Parkinson’s disease rating scale, PSPRS is the PSP rating scale. The scores for UPDRS, PSPRS and PSPRS stage are given untransformed, therefore a higher score indicates deteriorating function. t is the t statistic, df is the degrees of freedom, p values given are one tailed. P values are reported uncorrected. The p value for which differences in baseline and interval scores would be significant using the Bonferroni correction controlling for 3 comparisons is p<0.017. The UPDRS and PSPRS but not the PSPRS stage are significant at this level (marked with an asterisk *).

**Table 5:** Baseline and interval scores with paired t tests for cognitive tests.

	**Brixton**	**FAB**	**Hayling A**	**Hayling B**	**ACE-R**
**Baseline**	2.8	11.7	12	12.5	79.7
**Interval**	2.7	11.7	11.9	12.9	76.6
**t (df)**	0.3 (15)	0.4 (14)	0.08 (15)	-0.7 (15)	1.9 (15)
**p value**	0.8	0.7	0.9	0.2	0.04

Mean baseline and interval scores are given. FAB is the frontal assessment battery, Hayling A is the number of category A errors in the Hayling test, and Hayling B the number of category B errors. ACE-R is the Addenbrooke’s Cognitive Examination Revised. The scores for Hayling A errors and Hayling B errors are given untransformed, therefore a higher score indicates more errors and therefore deteriorating function. t is the t statistic, df is the degrees of freedom, p values given are one tailed. The p value for which differences in baseline and interval scores would be significant using the Bonferroni correction for 5 comparisons is p<0.01. None of the cognitive tests are significant at this level.

**Table 6:** Baseline and interval scores with paired t tests for visual and oculomotor assessment tests.

	**VOSP**	Mu*	**Sigma**
**Baseline**	8.9	4.3	1.4
**Interval**	8.7	3.9	1.4
**t (df)**	0.6 (15)	2.5 (13)	0.4(13)
**p value**	0.3	0.01	0.4

Mean baseline and interval scores are given. VOSP is the Visual Object and Space Perception battery, mu is the mean of the reciprocals of the median latency as measured by saccadometry, sigma is the mean of the variance of the saccade latencies. t is the t statistic, df is the degrees of freedom, p values given are one tailed. The p value for which differences in baseline and interval scores would be significant using the Bonferroni correction for 3 comparisons is p<0.017. Only mu is significant at this level (marked with an asterisk*).

Although the comparisons between baseline and interval only included patients for whom we had both baseline and interval data, we looked for differences between those baseline patients who could not complete interval testing and those who could, in case their drop out had caused a bias. Demographically, there was no difference between these two patient subgroups in age (*t*(37)=0.4, *p*=0.7), gender (χ^2^(1)=0.01, *p*=1.0), education years (*U*=183, *p*=1.0) or disease duration (*U*=115, *p*=0.05). The lack of progression of cognition over a year was surprising, so we also looked at cognitive differences at baseline between those patients who we could and couldn’t test at interval. Allowing for Bonferroni corrections (p<0.01 as significant for 5 comparisons) there were no differences (FAB (t(19)=1.7, p=0.12), Hayling A (t(21)=0.1, p=0.9), Hayling B (t(21)=0.9, p=0.4), ACE-R (t(21)=2.4, p=0.03, Brixton (t(18)=-1.3, p=0.2)).

**Figure 5:**
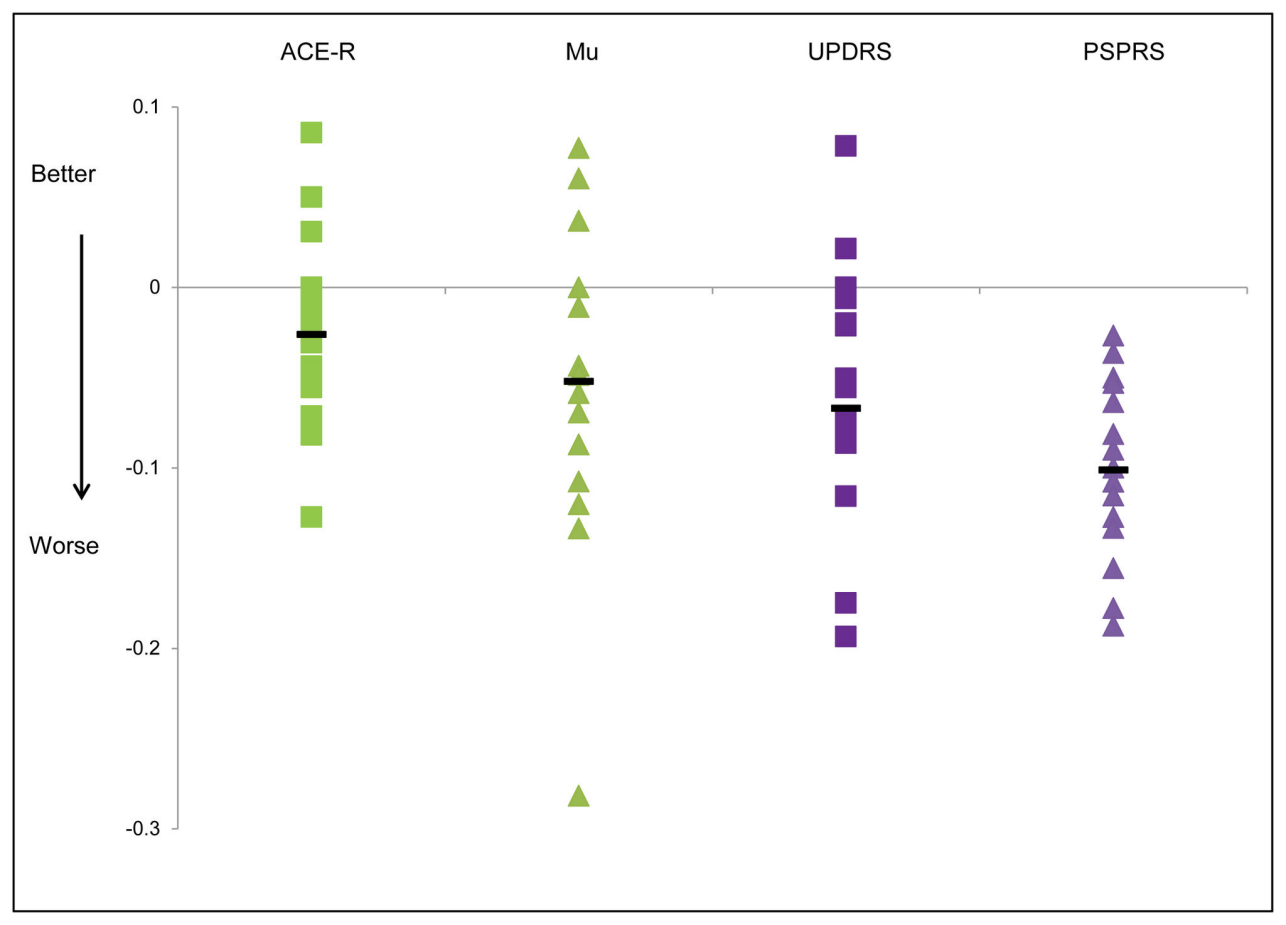
Graph showing annualised and normalised rates of change for different aspects of disease. Green squares are the change in scores for the Addenbrookes Cognitive Examination Revised, green triangles are rates of change for mu (reciprocal latency), purple squares are the change in scores for the Unified Parkinson’s Disease Rating Scale (UPDRS) motor subsection and the purple triangles are the change in scores for the PSP rating scale (PSPRS). The horizontal black lines are the mean rate of change for each test. Values below the x axis represent worsening of patients’ conditions and those above the x axis are improving.

Figure 5 shows the annualised normalised rate of change of the UPDRS, PSPRS, mu and the ACE-R. Normalising each test to its maximum enables comparison directly across tests. It can be seen that cognitive function as measured by the ACE-R changes only very slightly over a year whereas the PSPRS changes markedly. Repeated measures ANOVA for the normalised annualised rates of change for ACE-R, UPDRS, mu and PSPRS shows that there was a significant main effect for rates of change in different domains of the PSP phenotype (*F*(3,39)=3.15, *p*=0.036). Post hoc contrasts revealed that rates of change were significantly more for PSPRS when compared to ACE-R (*F*(1,13)=17.2, *p*=0.001) but not for mu compared to PSPRS (*F*(1,13)=3.4, *p*=0.09) or for ACE-R compared to UPDRS (*F*(1,13)=3.9, *p*=0.7).

**Figure 6:**
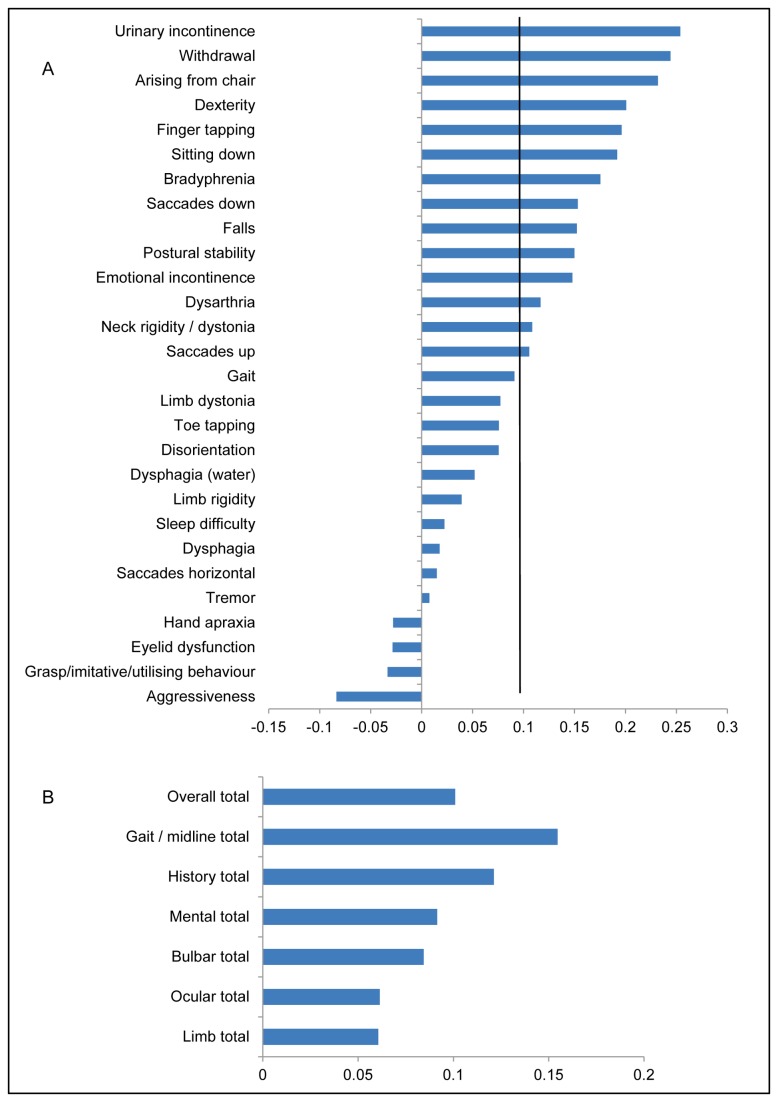
Graph showing the rate of change for individual items (A) and section totals (B) of the PSP rating scale (PSPRS). Bars extending to the right of the y axis are those parts of the scale where there has been a worsening of symptoms, those to the left an improvement of symptoms. The scale on the x axis refers to annualised normalised rates of change. The black vertical line on Figure A corresponds to the mean change of the overall total of the PSPRS scale.

Figure 6A shows how each element of the PSPRS changed over the course of a year, ordered from most change to the least. Figure 6B shows the subsection scores of the PSPRS ordered by most change. As can be seen in Figure 6, the gait/midline sections of the scale undergo the most change, followed by changes expressed in the history given by the carer and patient.

We extended the analyses to investigate the potential role of the tests used in this study in therapeutic trials. The change in test scores over an interval of a year was used to calculate an effect size for power calculations. Table 7 shows the estimated group sizes needed to reveal a putative reduction of 25 and 50% in the rate of decline on each of the principal measures, over 12 months.

**Table 7:** Sample size estimates for different markers of PSP progression.

**Test**	**d**	**Group size 25% effect**	**Group size 50% effect**
**PSPRS**	1.2	176	45
**UPDRS**	0.73	486	119
**PSPRS stage**	0.51	967	243
**Mu**	0.45	1,242	312
**ACE-R**	0.27	3,447	863
**Brixton**	0.07	51,259	>12,000
**Hayling A**	0.02	627,910	>150,000
**FAB**	0.01	2,511,637	>620,000

The group size is estimated for each arm of an intervention (not including attrition) where a therapy reduced progression of that aspect of the disease by 25 or 50%. Alpha is set at 0.05, beta (β) at 0.2 (ie. a power of 80%). PSPRS is the progressive supranuclear palsy rating scale, UPDRS the motor section of the unified Parkinson’s disease rating scale, Mu is the reciprocal of the latency of visually evoked horizontal saccades, ACE-R is the Addenbrooke’s Cognitive Examination Revised, Brix is Brixton, Hay A is the number of type A errors in the Hayling test and FAB is the Frontal Assessment Battery.

In addition, we investigated the correlation between baseline test scores and disease duration. Examining the patients for whom we had both baseline and interval data, there was a significant correlation between PSPRS and disease duration (Pearson correlation 0.68, p (2 tailed) = 0.005), with none of the other 11 measures tested having a p value of less than 0.1. Using all patients tested at baseline, PSPRS trended to a correlation (Pearson correlation 0.428, p=0.06). When we looked at correlations between annualised rate of change and disease duration, only 2 measures had a significance with p<0.1: Sigma (Pearson correlation 0.64, p=0.008) and PSPRS which trended to significance with Pearson correlation 0.449, p=0.081. However, none of these were significant if Bonferroni corrections were used (p<0.004).

## Discussion

This study examined the longitudinal change in progressive supranuclear palsy, including the cognitive, motor and oculomotor dimensions of this complex disease. Over one year, motor functions, and oculomotor decision time (saccade latency) changed significantly. However, multiple cognitive measures did not change significantly, despite being profoundly affected by the presence of disease at baseline.

The test exhibiting the greatest annual change was the PSP rating scale (see Figure 5) [5]. Other studies have also shown a comparable change in this composite score [4–6]. Golbe et al. showed an annual change of 11.3 points per year, Payan et al. 8.7 points per year and Whitwell et al. showed a change of 18 points. Whitwell et al. went on to consider the change in PSPRS and the change in volume of the midbrain, measured using MRI, as biomarkers for future trials. For 80% power with a 40% reduction in outcome variable over a year, 36 patients were estimated to be needed in each treatment arm using midbrain volume and 45 for total PSPRS score.

One of the most interesting and novel results is that cognition, despite being very disordered in a significant proportion of patients, does not change appreciably over the course of a year (see Table 5 and Figure 3). This stability was seen in multiple tests, including the ACE-R, Hayling test errors, Frontal Assessment Battery and Brixton test. The only exception was the mental sub section of the PSPRS (see Figure 6). This section of the PSPRS has a measure of bradyphrenia – a cardinal cognitive feature of PSP – which deteriorated over one year even though it is not objectively measured or operationalised.

Cross sectional studies have found no correlation between cognitive function and disease duration [20–22], supporting our findings. However, some studies have reported that cognitive symptoms appear to progress throughout the disease [1,4,6,23–27]. Only three of these were prospective longitudinal studies, of which, one used the PSPRS [4] and one used, as in the PSPRS, a carer reported questionnaire biased towards apathy, bradyphrenia and depression [6]. The other longitudinal study [24] found only a 12% increase in frontal lobe symptomatology between first and last visit (46% to 58%) compared to larger increases in other features of the disease, suggesting cognitive impairment had developed early and then remained relatively stable. Early cognitive impairment is also suggested by a PET study which found frontal cortical hypometabolism in patients with mild disease [28]. Taken together, these data could imply that frontal cognitive dysfunction occurs before overt presentation of the motor symptoms that usually lead to diagnosis and then remains relatively stable, apart from bradyphrenia. New treatments to avert cognitive impairment in PSP would therefore require a transformation in the awareness, recognition and specialist referral pathways for PSP.

Within the PSPRS, it can be seen (Figure 6) that large changes occur for the gait/midline and history sections. Early falls are a core inclusion criteria for PSP, while the presence of a gait/midline disorder is reflected in the supportive criteria [8]. Although progression often leads to wheelchair dependence, other midline features (neck rigidity for example) appear to continue to progress. The change in gait/midline problems on the PSPRS is mirrored in the UPDRS motor section and has been seen in other studies [4,6].

The change in PSPRS mean score for dysphagia was below the rate of change of the overall score and that for dysarthria is only just above the overall score. Several studies have shown that the leading cause of death in PSP is respiratory complications arising from aspiration [24,29–31], with dysarthria also deteriorating during the illness [6,24,25,30,32]. We may have seen greater changes if our patients had been at more advanced stages of the illness. However, these findings may also be due to dysarthria and dysphagia being variable during the course of the day or non-linearity of the scale, relying, for example, on the patient coughing once or many times on drinking water [5].

Oculomotor function changed over one year, including the range of vertical gaze in the PSPRS (cf [33]). Horizontal gaze range, in contrast, was stable. However, we were especially interested in the latency immediately prior to a saccade, resulting from a cortical and subcortical supranuclear oculomotor decision network. We identified a small but significant change in latency (mu, reciprocal latency) between baseline and interval, indicating an increase in latency for visually evoked horizontal saccades. Such saccades have been proposed as a biomarker for the diagnosis or progression of PSP and other neurodegenerative disorders [34–37], although other studies have found variable changes in saccade parameters over time [6,37,38]. We suggest that while the latency of horizontal saccades remains useful to explore the neural systems of decision making in disease, and perhaps long term change in PSP, it is not optimal as a marker of change over 1 year, the typical timescale for pharmaceutical trials.

In our study, the greatest change was seen in the PSPRS, particularly the gait/midline section. We did not assess the Parkinson’s plus scale developed within the NNIPPS trial (the Natural history and Neuroprotection in Parkinson Plus Syndromes, NNIPPS-PPS) [6]. This scale was developed by a consensus of experts and includes sections similar to parts of the UPDRS (motor, mental and activities of daily living (ADL) sections) and the PSPRS (mental section). As in our study, the greatest annual change was seen in mobility, axial bradykinesia and rigidity. They also found a large annual change in limb bradykinesia, whereas our limb section of the PSPRS was one of the sections that changed least.

By contrasting the range of tests which are abnormal at baseline in PSP (median time from onset to recruitment 3.0 years) with the tests which change over one year’s interval, it is clear that motor, oculomotor and cognitive systems evolve at different timescales. From our data, cognition deteriorates early in susceptible individuals and then remains relatively stable. Motor deficits also occur early but continue to progress throughout the whole course of the disease. Oculomotor function, in terms of the range of eye movements, deteriorates before the time of diagnosis but continues to progress slowly during the middle stage of disease. Oculomotor function in terms of saccadic latency may be affected by disease, and may be informative about within-subject differences in cognition or brain function [36] but does not change markedly at the group level.

The differential rates of deterioration may be due to the relatively domain specific cortical – subcortical pathways [39] having different susceptibilities to PSP pathology or different capacities for functional compensation. This differential progression has important implications for patients. However it is also relevant to disease-modifying drug trials, where it is critical to choose the best marker of disease progression. We have used our data to assess the tests used as markers of disease progression, giving sample sizes needed depending on the effect size and likely effect of any treatment (table 7). It should be noted that our calculations pertain to studies of comparable patients at similar stages of disease (see tables 2 and 3 for our baseline data), and inclusion of early stage patients may lead to different power calculations. However, we recruited prospectively from patients who presented to a regional clinic with a diagnosis of PSP during the time of the study, it is likely that our cohort is typical of many other centres. It should also be borne in mind that symptomatic disease duration is not a good proxy for stage. Although in our sample, the estimated duration did correlate with baseline PSPRS score, it did not significantly correlate with rate of change of PSPRS or other baseline motor and cognitive measures. We also note that one of our patients had relatively slow progression, surviving 17 years. This is within diagnostic criteria and within the range of published cohorts, but nonetheless unusual.

Using these power calculations, the group sizes vary widely depending on the different elements of the disease that investigators may want to influence. Assessing improvement in cognition would require over 800 patients in each group using the ACE-R [10] but assessing global function requires a more tractable 45 patients using the PSPRS [5], which accords with the estimate from Whitwell et al. [4]. Payan et al. also assessed the PSPRS and estimated 100 patients in each group would be needed, but only 40 if using the NNIPPS-PPS [6].

There are several potential limitations to our study. Firstly, inclusion criteria relied on clinical rather than pathological diagnosis. However, ten cases have subsequently had a post mortem examination and the diagnosis of PSP was confirmed in all ten. Larger trials have also shown a diagnostic accuracy in excess of 90% [7]. Secondly, our study is relatively small. However, patients had typical clinical phenotypes of PSP (cf Richardson’s syndrome, PSP-RS) and completed an intensive evaluation across many functional domains over one year, providing a significant addition to the previous literature on cognitive, motor or oculomotor progression. PSP usually progresses quickly and it is possible that those patients who did not complete interval testing represented those who had a more aggressive phenotype of the disease, leaving behind a subset of patients who were less likely to change over a year. Against this possibility however, is that there was no difference between survivors and non-survivors in terms of demographics, cognition or disease duration to suggest that they represented distinct populations. Furthermore, comparisons between baseline and interval metrics were only carried out between patients who had completed both sets of tests.

Another significant limitation is that patients may have different rates of decline for different functions as the disease progresses (eg. cognitive vs motor). Furthermore, tests may vary in their ability to represent the true function of patients through the course of the illness e.g. due to floor or ceiling effects. The interpretation and replication of our data, including power calculations for interventional trials, should therefore take into account the baseline characteristics of our cohort, including stage or severity of disease.

In conclusion, we suggest that cognition does not change appreciably over a year in the middle stages of PSP (after diagnosis), a novel result that may shed light on the underlying pathological deterioration in PSP and which needs to be replicated in further studies. Of clear significance to the development of new treatments, is that we have shown that patients show significant deterioration over one year using the PSPRS severity measure. Indeed, including patients who were clinically diagnosed with PSP [5], which is broadly equivalent to the operational diagnostic criteria used by a recent trial [7], annual change in the PSP rating scale was matched between our study and the original PSPRS study, at 11.3 points a year [5]. We also concur with Whitwell et al. that using the PSPRS, approximately 45 patients in each treatment arm would provide reasonable power for future clinical trials of a highly effective treatment (50% slowing of annual decline) [4]. The high rate of pathological confirmation from the PSP clinical phenotype [7] and the properties of the PSPRS, support the use of these simple tools in new clinical trials of this devastating disease.

## References

[1] Steele JC, Richardson JC, Olszewski J (1964) Progressive Supranuclear Palsy. a Heterogeneous Degeneration Involving the Brain Stem, Basal Ganglia and Cerebellum with Vertical Gaze and Pseudobulbar Palsy, Nuchal Dystonia and Dementia. Arch Neurol 10: 333–359. doi:10.1001/archneur.1964.00460160003001. PubMed: 14107684. 14107684 10.1001/archneur.1964.00460160003001

[2] Gold M, Lorenzl S, Stewart AJ, Morimoto BH, Williams DR et al. (2012) Critical appraisal of the role of davunetide in the treatment of progressive supranuclear palsy. Neuropsychiatr Dis Treat 8: 85–93. doi:10.2147/NDT.S12518. PubMed: 22347799. 22347799 10.2147/NDT.S12518 PMC3280109

[3] Paviour DC, Price SL, Lees AJ, Fox NC (2007) MRI derived brain atrophy in PSP and MSA-P. Determining sample size to detect treatment effects. J Neurol 254: 478–481. doi:10.1007/s00415-006-0396-4. PubMed: 17401522. 17401522 10.1007/s00415-006-0396-4

[4] Whitwell JL, Mandrekar JN, Gunter JL, Jack CR Jr, Josephs KA (2012) Rates of brain atrophy and clinical decline over 6 and 12 month intervals in PSP: determining sample size for treatment trials. Parkinsonism Relat Disord 18: 252–256. doi:10.1016/j.parkreldis.2011.10.013. PubMed: 22079523. 22079523 10.1016/j.parkreldis.2011.10.013 PMC3399183

[5] Golbe LI, Ohman-Strickland PA (2007) A clinical rating scale for progressive supranuclear palsy. Brain 130: 1552–1565. doi:10.1093/brain/awm032. PubMed: 17405767. 17405767 10.1093/brain/awm032

[6] Payan CAM, Viallet F, Landwehrmeyer BG, Bonnet A-M, Borg M et al. (2011) Disease Severity and Progression in Progressive Supranuclear Palsy and Multiple System Atrophy: Validation of the NNIPPS – PARKINSON PLUS SCALE. PLOS ONE 6: e22293. doi:10.1371/journal.pone.0022293. PubMed: 21829612. 21829612 10.1371/journal.pone.0022293 PMC3150329

[7] Bensimon G, Ludolph A, Agid Y, Vidailhet M, Payan C et al. (2009) Riluzole treatment, survival and diagnostic criteria in Parkinson plus disorders: the NNIPPS study. Brain 132: 156–171. PubMed: 19029129. 19029129 10.1093/brain/awn291 PMC2638696

[8] Litvan I, Bhatia KP, Burn DJ, Goetz CG, Lang AE et al. (2003) Movement Disorders Society Scientific Issues Committee report: SIC Task Force appraisal of clinical diagnostic criteria for Parkinsonian disorders. Mov Disord 18: 467–486. doi:10.1002/mds.10459. PubMed: 12722160. 12722160 10.1002/mds.10459

[9] Fahn S (1986) Recent developments in Parkinson’s disease. New York: Raven Press. 375pp.

[10] Mioshi E, Dawson K, Mitchell J, Arnold R, Hodges JR (2006) The Addenbrooke’s Cognitive Examination Revised (ACE-R): a brief cognitive test battery for dementia screening. Int J Geriatr Psychiatry 21: 1078–1085. doi:10.1002/gps.1610. PubMed: 16977673. 16977673 10.1002/gps.1610

[11] Dubois B, Slachevsky A, Litvan I, Pillon B (2000) The FAB: a Frontal Assessment Battery at bedside. Neurology 55: 1621–1626. doi:10.1212/WNL.55.11.1621. PubMed: 11113214. 11113214 10.1212/wnl.55.11.1621

[12] Warrington EK, James M (1991) The visual object and space perception battery. Bury St. Edmunds, Thames Valley Test Company . 20 pp

[13] Burgess PW, Shallice T (1997) The Hayling and Brixton tests. Bury St Edmunds: Thames Valley Test Company. 20pp.

[14] Ober JK, Przedpelska-Ober E, Gryncewicz W, Dylak J, Carpenter RHS et al. (2003) Hand held system for ambulatory measurement of saccadic durations of neurological patients. Gajda J Warsaw: Komitet Biocybernityki i Inzyneierii Biomedycznej PAN. pp. 187-198.

[15] Carpenter R (1994) SPIC: a PC-based system for rapid measurement of saccadic responses. J Physiol (Proc) 480: 4P.

[16] Carpenter RHS, Williams MLL (1995) Neural Computation of Log Likelihood in Control of Saccadic Eye-Movements. Nature 377: 59–62. doi:10.1038/377059a0. PubMed: 7659161. 7659161 10.1038/377059a0

[17] Reddi BA, Asrress KN, Carpenter RH (2003) Accuracy, information, and response time in a saccadic decision task. J Neurophysiol 90: 3538–3546. doi:10.1152/jn.00689.2002. PubMed: 12815017. 12815017 10.1152/jn.00689.2002

[18] Erdfelder E, Faul F, Buchner A (1996) GPOWER: a general power analysis program. Behav Res Methods Instrum Comput 28: 1–11. doi:10.3758/BF03203630.

[19] Faul F, Erdfelder E (1992) GPOWER: A priori, post hoc and compromise power analysis for ms-dos [computer program]. Bonn, FRG: Bonn University, Department of psychology.

[20] Brown RG, Lacomblez L, Landwehrmeyer BG, Bak T, Uttner I et al. (2010) Cognitive impairment in patients with multiple system atrophy and progressive supranuclear palsy. Brain 133: 2382–2393. doi:10.1093/brain/awq158. PubMed: 20576697. 20576697 10.1093/brain/awq158

[21] Donker Kaat L, Boon AJ, Kamphorst W, Ravid R, Duivenvoorden HJ et al. (2007) Frontal presentation in progressive supranuclear palsy. Neurology 69: 723–729. doi:10.1212/01.wnl.0000267643.24870.26. PubMed: 17709703. 17709703 10.1212/01.wnl.0000267643.24870.26

[22] Ghosh BC, Rowe JB, Calder AJ, Hodges JR, Bak TH (2009) Emotion recognition in progressive supranuclear palsy. J Neurol Neurosurg, Psychiatry 80: 1143–1145. doi:10.1136/jnnp.2008.155846. PubMed: 19762901. 19762901 10.1136/jnnp.2008.155846 PMC3044450

[23] Albert ML, Feldman RG, Willis AL (1974) The “subcortical dementia” of progressive supranuclear palsy. J Neurol Neurosurg, Psychiatry 37: 121–130. doi:10.1136/jnnp.37.2.121. 4819905 10.1136/jnnp.37.2.121 PMC494589

[24] Litvan I, Mangone CA, McKee A, Verny M, Parsa A et al. (1996) Natural history of progressive supranuclear palsy (Steele: Richardson-Olszewski syndrome) and clinical predictors of survival: a clinicopathological study. J Neurol Neurosurg Psychiatry 60: 615–620. 8648326 10.1136/jnnp.60.6.615 PMC1073943

[25] Macia F, Ballan G, Yekhlef F, Delmer O, Vital C et al. (2003) Progressive supranuclear palsy: a clinical, natural history and disability study. Rev Neurol 159: 31–42. PubMed: 12618651. 12618651

[26] Pillon B, Dubois B (1992) Cognitive and behavioural impairments. In: Litvan I Agid Y Progressive supranuclear palsy : clinical and research approaches. New York: Oxford University Press.

[27] Santacruz P, Uttl B, Litvan I, Grafman J (1998) Progressive supranuclear palsy: a survey of the disease course. Neurology 50: 1637–1647. doi:10.1212/WNL.50.6.1637. PubMed: 9633705. 9633705 10.1212/wnl.50.6.1637

[28] D’Antona R, Baron JC, Samson Y, Serdaru M, Viader F et al. (1985) Subcortical Dementia. Brain 108: 785–799. doi:10.1093/brain/108.3.785. PubMed: 3876136. 3876136 10.1093/brain/108.3.785

[29] Maher ER, Lees AJ (1986) The Clinical-Features and Natural-History of the Steele-Richardson-Olszewski Syndrome (Progressive Supranuclear Palsy). Neurology 36: 1005–1008. doi:10.1212/WNL.36.7.1005. PubMed: 3714047. 3714047 10.1212/wnl.36.7.1005

[30] Müller J, Wenning GK, Verny M, McKee A, Chaudhuri KR et al. (2001) Progression of dysarthria and dysphagia in postmortem-confirmed Parkinsonian disorders. Arch Neurol 58: 259–264. doi:10.1001/archneur.58.2.259. PubMed: 11176964. 11176964 10.1001/archneur.58.2.259

[31] Papapetropoulos S, Singer C, McCorquodale D, Gonzalez J, Mash DC (2005) Cause, seasonality of death and co-morbidities in progressive supranuclear palsy (PSP). Parkinsonism Relat Disord 11: 459–463. doi:10.1016/j.parkreldis.2005.06.003. PubMed: 16154793. 16154793 10.1016/j.parkreldis.2005.06.003

[32] Nath U, Ben-Shlomo Y, Thomson RG, Lees AJ, Burn DJ (2003) Clinical features and natural history of progressive supranuclear palsy - A clinical cohort study. Neurology 60: 910–916. doi:10.1212/01.WNL.0000052991.70149.68. PubMed: 12654952. 12654952 10.1212/01.wnl.0000052991.70149.68

[33] Chiu WZ, Kaat LD, Seelaar H, Rosso SM, Boon AJW et al. (2010) Survival in progressive supranuclear palsy and frontotemporal dementia. J Neurol Neurosurg, Psychiatry 81: 441–445. doi:10.1136/jnnp.2009.195719. PubMed: 20360166. 20360166 10.1136/jnnp.2009.195719

[34] Ali FR, Michell AW, Barker RA, Carpenter RH (2006) The use of quantitative oculometry in the assessment of Huntington’s disease. Exp Brain Res 169: 237–245. doi:10.1007/s00221-005-0143-6. PubMed: 16273398. 16273398 10.1007/s00221-005-0143-6

[35] Michell AW, Xu Z, Fritz D, Lewis SJ, Foltynie T et al. (2006) Saccadic latency distributions in Parkinson’s disease and the effects of L-dopa. Exp Brain Res 174: 7–18. doi:10.1007/s00221-006-0412-z. PubMed: 16544135. 16544135 10.1007/s00221-006-0412-z PMC1877863

[36] Perneczky R, Ghosh BCP, Hughes L, Carpenter RHS, Barker RA et al. (2011) Saccadic latency in Parkinson’s disease correlates with executive function and brain atrophy, but not motor severity. Neurobiol Dis 43: 79–85. doi:10.1016/j.nbd.2011.01.032. PubMed: 21310235. 21310235 10.1016/j.nbd.2011.01.032 PMC3102178

[37] Pierrot-Deseilligny C, Rivaud S, Pillon B, Fournier E, Agid Y (1989) Lateral visually-guided saccades in progressive supranuclear palsy. Brain 112(2): 471–487. doi:10.1093/brain/112.2.471. PubMed: 2706440. 2706440 10.1093/brain/112.2.471

[38] Rivaud-Péchoux S, Vidailhet M, Gallouedec G, Litvan I, Gaymard B et al. (2000) Longitudinal ocular motor study in corticobasal degeneration and progressive supranuclear palsy. Neurology 54: 1029–1032. doi:10.1212/WNL.54.5.1029. PubMed: 10720270. 10720270 10.1212/wnl.54.5.1029

[39] Alexander GE, DeLong MR, Strick PL (1986) Parallel organization of functionally segregated circuits linking basal ganglia and cortex. Annu Rev Neurosci 9: 357–381. doi:10.1146/annurev.ne.09.030186.002041. PubMed: 3085570. 3085570 10.1146/annurev.ne.09.030186.002041

[40] Brusa A, Mancardi GL, Bugiani O (1980) Progressive supranuclear palsy 1979: an overview. Ital J Neurol Sci 1: 205–222. PubMed: 7338456. 7338456 10.1007/BF02336701

[41] Golbe LI, Davis PH, Schoenberg BS, Duvoisin RC (1988) Prevalence and Natural-History of Progressive Supranuclear Palsy. Neurology 38: 1031–1034. doi:10.1212/WNL.38.7.1031. PubMed: 3386818. 3386818 10.1212/wnl.38.7.1031

[42] Collins SJ, Ahlskog JE, Parisi JE, Maraganore DM (1995) Progressive supranuclear palsy: neuropathologically based diagnostic clinical criteria. J Neurol Neurosurg, Psychiatry 58: 167–173. doi:10.1136/jnnp.58.2.167. PubMed: 7876846. 7876846 10.1136/jnnp.58.2.167 PMC1073312

[43] Carrilho PEM, Barbosa ER (2002) Progressive supranuclear palsy in a sample of Brazilian population - Clinical features of 16 patients. Arq Neuro Psiquiatr 60: 917–922. doi:10.1590/S0004-282X2002000600006. 10.1590/s0004-282x2002000600006 12563380

[44] Goetz CG, Leurgans S, Lang AE, Litvan I (2003) Progression of gait, speech and swallowing deficits in progressive supranuclear palsy. Neurology 60: 917–922. doi:10.1212/01.WNL.0000052686.97625.27. PubMed: 12654953. 12654953 10.1212/01.wnl.0000052686.97625.27

[45] O’Sullivan SS, Massey LA, Williams DR, Silveira-Moriyama L, Kempster PA et al. (2008) Clinical outcomes of progressive supranuclear palsy and multiple system atrophy. Brain 131: 1362–1372. PubMed: 18385183. 18385183 10.1093/brain/awn065

